# Recent Advances in Scaffolding from Natural-Based Polymers for Volumetric Muscle Injury

**DOI:** 10.3390/molecules26030699

**Published:** 2021-01-29

**Authors:** Tamrin Nuge, Ziqian Liu, Xiaoling Liu, Bee Chin Ang, Andri Andriyana, Hendrik Simon Cornelis Metselaar, Md Enamul Hoque

**Affiliations:** 1 Department of Mechanical, Materials and Manufacturing Engineering, Faculty of Science and Engineering, University of Nottingham Ningbo China, 199 Taikang East Road, Ningbo 315100, China; tamrin.nuge@nottingham.edu.cn (T.N.); ziqian.liu@nottingham.edu.cn (Z.L.); 2 Centre of Advanced Materials, Faculty of Engineering, University of Malaya, Kuala Lumpur 50603, Malaysia; andri.andriyana@um.edu.my (A.A.); h.metselaar@um.edu.my (H.S.C.M.); 3 Department of Chemical Engineering, Faculty of Engineering, University of Malaya, Kuala Lumpur 50603, Malaysia; 4 Department of Mechanical Engineering, Faculty of Engineering, University of Malaya, Kuala Lumpur 50603, Malaysia; 5 Department of Biomedical Engineering, Military Institute of Science and Technology (MIST), Dhaka 1216, Bangladesh; enamul1973@gmail.com

**Keywords:** Volumetric Muscle Loss (VML), tissue engineering, electrospun, hydrogels, acellular, stem cells

## Abstract

Volumetric Muscle Loss (VML) is associated with muscle loss function and often untreated and considered part of the natural sequelae of trauma. Various types of biomaterials with different physical and properties have been developed to treat VML. However, much work remains yet to be done before the scaffolds can pass from the bench to the bedside. The present review aims to provide a comprehensive summary of the latest developments in the construction and application of natural polymers-based tissue scaffolding for volumetric muscle injury. Here, the tissue engineering approaches for treating volumetric muscle loss injury are highlighted and recent advances in cell-based therapies using various sources of stem cells are elaborated in detail. An overview of different strategies of tissue scaffolding and their efficacy on skeletal muscle cells regeneration and migration are presented. Furthermore, the present paper discusses a wide range of natural polymers with a special focus on proteins and polysaccharides that are major components of the extracellular matrices. The natural polymers are biologically active and excellently promote cell adhesion and growth. These bio-characteristics justify natural polymers as one of the most attractive options for developing scaffolds for muscle cell regeneration.

## 1. Introduction

Skeletal muscle dysfunctions can be caused by acquired disorders of muscle (e.g., inflammation, toxic, endocrine), genetic (e.g., muscular dystrophy or congenital myopathy), cachexia (e.g., HIV, cancer, chronic obstructive pulmonary disease (COPD)), sarcopenia or injury [1,2]. Skeletal muscle has high regeneration potential after tissue damage due to the presence of satellite cells. The satellite cell provide the skeletal muscle with a remarkable capacity for rapid and repeated repair and regeneration [3]. However, rapid regeneration of skeletal muscle is limited to acute and chronic muscle injuries and is not applicable for the case of volumetric muscle loss (VML) injury (substantial skeletal muscle tissue loss either through trauma or surgery resection) [4]. The VML injury exhibits a defect region where all key elements involved as a regenerative cue such as basal lamina and satellite cells are missing. Hence, the tissue manifests poor regeneration capacity [5]. The formation of non-contractile scar tissue at the defect site has caused the patient to manifest functional disability [6]. The current standard of care entails the use of muscle pedicle flap isolated from adjacent injury’s regions [7]. However, the standard care is always hindered by the host muscle tissue availability and donor site morbidity due to poor strength of the underlying muscle flaps [8]. Ineffective treatment options with poor clinical outcomes in conventional VML treatment strategies motivates our exploration, in order to provide the reader with the most current strategies and effective therapeutic approach on VML treatment. 

Advances in material engineering and known chemistry of synthetic materials including greater accessibility to control chemical modification have triggered the development of engineered polymer-based scaffolds for the use in various biomedical fields including treatment of VML. Synthetic polymers are an attractive class of materials in the biomedical fields, especially in tissue scaffolds, due to their useful traits and can be easily tailored to meet specific mechanical properties, porosity and degradation time according to their application requirements. In recent years, major classes of biodegradable polymers such as polyesters, polyurethane and polyamides are among the commonly used polymers in tissue engineering, due to the predictable and reproducible mechanical and physical properties such as tensile strength, elastic modulus and degradation rate. Moreover, synthetic polymers are well known for their enormous availability and can be easily mass-produced with uniform quality at a low cost. However, despite the excellent qualities offered by the synthetic polymers, concerns about the toxicity always hindered their employability as implants in the human body. The toxicity from implants degradation is always associated with the release of polymer degradation products and by-products in a local acidic environment at the implant site [9]. The small particles from the polymer degradation could trigger an inflammatory response especially when captured in a confined space [10]. To date, many studies have been carried out to synthesize novel bio-based materials by employing non-harmful natural polymers, such as palm oils and soybean oil via solvent-free polymerization techniques to reduce the toxicity resulting from the solvent impurity and formation of residual monomers during degradation. 

Compared with its synthetic counterparts, natural polymers are often components in the extracellular matrix (ECM), a macromolecular network present within cells. This makes natural polymer is mostly biocompatible with less inflammatory response, hence providing a conducive environment for cells proliferation. Attempts have been made to use scaffolds composed of natural polymers to promote the repair of VML injury by providing structural and biochemical frameworks. The natural polymer-based scaffold properties are expected to closely resemble those of the ECM macromolecular network that offers mechanical stability and structural integrity to tissue and organ. The inherent capacity for cell binding is exerted by the presence of the following molecular sequences: Polypeptide base—carrying specific protein structural motif such as RGD (arginine/Glycine/aspartic acid) [11] and polysaccharides base—numerous hydroxyl groups that provide multiple sites for the attachment of side groups [12]. 

The present review provides a short overview of skeletal muscle structure and organization, volumetric muscle loss along with recent advances in tissue engineering scaffolds to treat the VML. The cell source and critical design criteria for developing scaffolds for In vitro cell expansion are highlighted. Finally, future direction and challenges in the field of severe skeletal muscle injury are examined.

## 2. Structure and Organization of Skeletal Muscle Tissue

**Figure 1:**
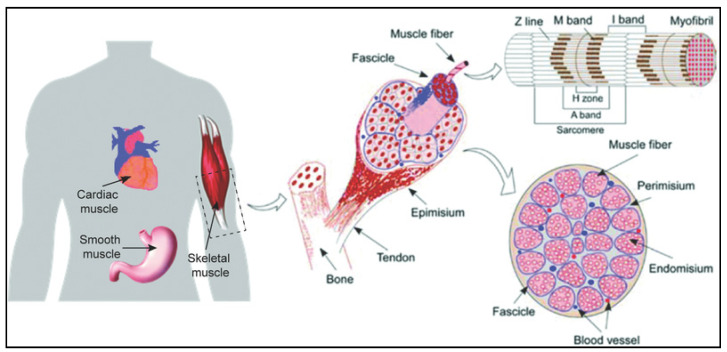
Schematic illustration of anatomic structures and organization of skeletal muscle tissue [17]. Copyright © 2016 by John Wiley and Sons. Reprinted by permission of John Wiley and Sons.

Skeletal muscle also known as voluntary muscle is a biological machine that converts the adenosine triphosphate (ATP), the source of energy used for force production and mechanical work [13]. The human body is composed of approximately around 640 skeletal muscles and almost all are in pairs, which comprised almost 38%, and 30% of total body mass for men and women, respectively [14]. Each skeletal muscle is made up of thousands of long cylindrical multinucleated cells, mitochondria and sarcomeres known as muscle fiber or myofiber. The skeletal muscles also consist of various integrated tissues including connective tissues, blood vessels and nerves (Figure 1) [15]. Skeletal muscle has a remarkable capacity to regenerate with a rapid re-establishment estimated within 21 days and capable of re-innervate even after repeated injury [16]. The source for the remarkable regenerative capacity of the skeletal muscle is guaranteed by the satellite cells. These cells can fuse or damage muscle fibers to regenerate and repair the damage fibers [15]. However, in the case of traumatic loss or surgical removal of muscle tissue (>20% by mass), the regenerative cues provided by satellite cells are missing, thus, such skeletal muscle tissue undergoes fibrosis, the replacement of muscle fibers by fibrous scar tissue [6]. These injuries result in chronic functional limitations and further deteriorate over time 

## 3. Volumetric Muscle Loss (VML)

**Figure 2:**
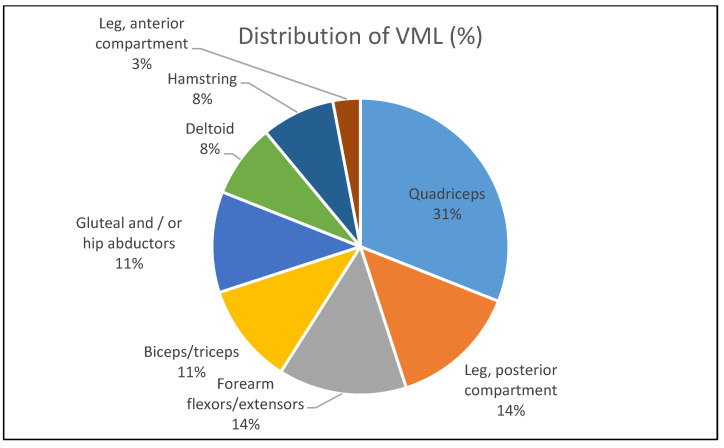
Distribution of VML per body part for a general cohort of 36 patients. The distribution of VML per body part was derived from the literature review [8].

Major traumatic injuries involved severe damage of skeletal muscle and peripheral nerves often impaired the function of myoneural junctions, chemical communication between a nerve fiber and a muscle cell [18]. Upon skeletal muscle injury, the muscle regeneration cascade would be activated. The complex pathway’s cascade would regulate satellite cell proliferation and differentiation into multinucleated myotubes that eventually differentiate into mature fibers at the damage site. However, the capacity of satellite cell declines with aging. The cell’s competency is diminished after large volumetric muscle loss as the cells are finite. This phenomenon has been shown to induce the formation of robust scar and loss of function attributed to the changes in muscle architecture and composition. Since no suitable therapies are clinically available thus far, VML is often left untreated and considered part of the natural sequelae of severe musculoskeletal trauma [7]. The untreated VML injury would cause patients to lose their quality of life due to significant loss of muscle strength and limb function attributable to extensive fibrosis [19]. According to Corona and co-workers [8], VML injury contributes to more than 90% of muscle conditions that lead to long term disability among the battlefield injured service members in the United States. Most of the patients reported in the study were suffered from permanent loss of limb function. Immediate intervention can minimize the damage. Corona et al. [6] reported that earlier therapeutic intervention in an animal model with VML injury shows positive results on the motor unit expansion believed to support muscle regeneration. Hitherto, limited clinical data on motor unit expansion on VML injury’s patients as the clinical repair only took part at least a year after post-injury. Figure 2 depicts the distribution of VML per body part for the general cohort as described by Corona and colleagues. 

## 4. Tissue Engineering Approaches for VML

The current gold standard of care for VML is typically based on surgical intervention with autologous muscle graft. This procedure involves a healthy muscle transplant from an unaffected site to restore the loss of impairment functions. When no adjacent muscle is available, autologous transplantation and neurorrhaphy in the form of free functional muscle transfer would be applied [20]. However, shortage of human organ donors and additional health risks frequently associated with the procedure. The deforming donor site morbidity and chronic pain during healing should also be considered when opting for muscle grafting. Statistically, almost 10% of graft failure is due to complications such as venous thrombosis [21], arterial occlusion [22], infection and mechanical stress around the anastomosis [23]. In recent years, tissue engineering has presented a promising approach to address these challenges by providing new sources of tissues and enabling angiogenesis into the tissues after implantation [24]. In the following sections, new approaches for VML defect repairs by tissue engineering approaches are discussed. 

### 4.1. Cell Based Therapies for Muscle Injury

**Figure 3:**
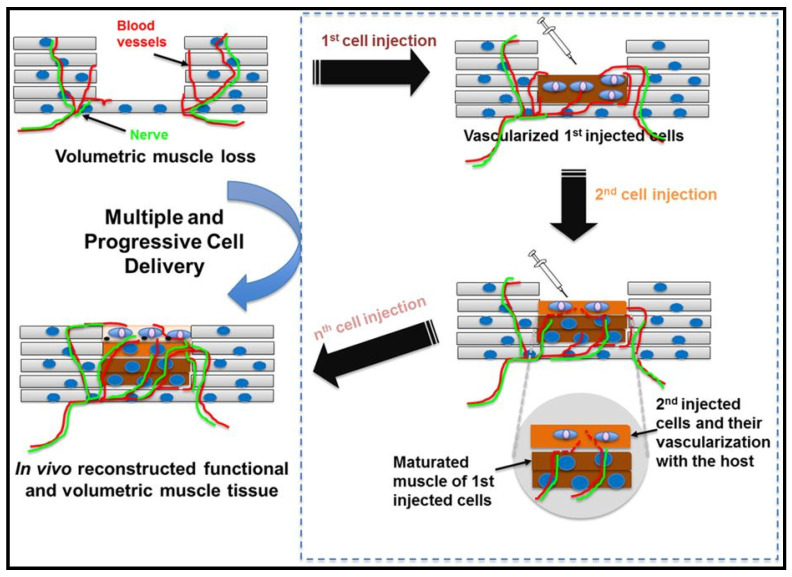
Schematic illustration of cell-based therapy for muscle cell delivery as a solution for volumetric muscle repair [4]. Reprinted by permission of springer Nature. Copyright © 2016 by Springer Nature.

Cell based-therapies represent the most exciting and promising strategies to restore normal functions of damaged and injured tissues and organs [4]. The strategies involve transplantation and implantation of cells derivatives or engineered tissue construct into the affected region. Stem cells display some characteristics that make them a favourable cell source in therapeutic muscle injury. Along with inherent self-renewal and differentiation potential, stem cells can be manipulated for cells source in various therapies [25]. The stem cells can also easily migrate and differentiate at the injury site and release paracrine signaling cascade such as growth factors that involve tissue repair [26]. In the affected regions, the stem cells can be delivered via intravenous injection (Figure 3), direct transplantation or isolated from patient’s tissues for self-repair induction. In the remainder of this section, recent advances of stem cells as cell-based therapy are discussed details. 

#### 4.1.1. Embryonic Stem Cells (ESCs)

Embryonic stem cells (ESCs) are stem cells derived from the undifferentiated inner cell mass of human embryos [27]. ESCs are pluripotent as they can grow indefinitely and differentiate into all derivatives of three primary germ layers. ESCs have been proposed as an attractive and viable proposition for cell replacement therapy including VML. The characteristics offered by ESCs such as developmental plasticity, potentially unlimited capacity for self-renewal and the ability to be induced to differentiate into different lineages make them the best candidate to offset impaired healing [28]. Caspi et al. [29] demonstrated that ESCs seeded on 3D biodegradable highly porous polymeric scaffolds had improved tissue vascularization and regeneration, which may further enhance tissue graft function and survival. Despite their tremendous potential in tissue regeneration, no approved medical treatment has been derived from embryonic stem cells research, thus far. Cananzi et al. [30] listed the major constraints of ESCs that inhibit their deployment in tissue regeneration; (i). Ethical issues/controversy concerning their isolation from human embryos. (ii). Safety concerns regarding their observed tendency to form tumors when injected undifferentiated or only partially differentiated in vivo. (iii). Possible host immune rejection of cellular allografts. All of these drawbacks hindered the exploration of using ESCs in cell therapy.

#### 4.1.2. Adult Mesenchymal Stem Cells (MSCs)

**Figure 4:**
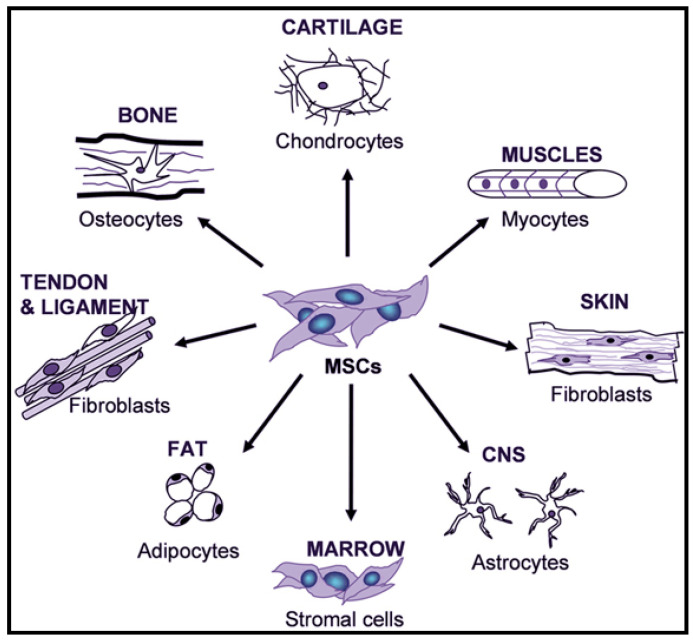
Multi-lineage potential of MSCs showing the potential to differentiate into various lineages making them the ideal candidates for cell-based tissue engineering strategies [34]. Copyright © 2018 by Frontiers in Bioscience. Reprinted by permission of Frontiers in Bioscience.

Ethical tensions related to ESCs clinical translation have limited the use of embryonic stem cells as a cell-based therapy in regenerative medicine. This makes the mesenchymal stem cells (MSCs) an appealing choice in clinical translation as the MSCs are self-renewal, well-tolerated and have no ethical concerns [31]. Mesenchymal stem cells are heterogenous multipotent stem cells that can be isolated from the placenta, peripheral blood, bone marrow, umbilical cord, and adipose tissue [26]. The stem cells have been well characterized to be multipotent cells that can differentiate into multiple tissue-forming cell lineages, such as keratinocytes, adipocytes, etc. (Figure 4) [32]. MSCs are reported to regulate VML repair through the excretion of paracrine signaling cascade, such as growth factors confirmed to contribute to myofiber formation and functional recovery of muscle tissue [33]. The active role of mesenchymal stem cells in maintaining satellite cell activity, by suppressing myonuclear apoptosis, enables their use in treating VML defects. 

The earliest work on the employment of MSCs isolated from bone marrow to treat transgenic mice’s injured muscle was first reported by Ferrari et al. [35]. They found that the MSCs isolated from bone marrow progressively participated in the regenerative process and replaced the exhausted pool of satellite cells. To further study the involvement of the MSCs derived from bone marrow on muscle repair, LaBarge and Blau [36], reported that the adult MSCs isolated from bone marrow were capable of migrating and differentiating into satellite cells at the muscle fibers after trauma induced-damage. This suggested that the MSCs isolated from bone marrow could potentially serve new therapies in fighting against diseases, as well as a back-up source in damaged tissues reparative. Attempts have been made to investigate the efficacy of MSCs aspirated from bone marrow on severe muscle injury. Winkler et al. [37] have found that the MSCs are promoting muscular regeneration, but a large number of MSCs populations are required to trigger the cascade healing. They had established that the progress of muscle contraction force depends on the number of MSCs injected at the injury site. In other studies, they had conducted the best timing for MSCs transplantation for muscle injury [38]. No significant difference was observed in muscle regeneration functionality between immediate and seven days delayed MSCs transplantation after injury. Both treatment groups manifested MSCs residing in the interstitial compartment rich with extracellular matrices that regulate the paracrine pathways. Despite their promising results, much work remains to be done before MSCs can pass from the bench to the bedside for treating muscle injuries, including VML. Poor cell retention and differentiation due to the harsh microenvironment at the injury site remain the major challenge in applying the MSCs. Ferrari and Mavilio [39] demonstrated the implanted MSCs on muscular dystrophy mice exhibited poor contribution to dystrophin formation due to a lack of signals from the muscles of the dystrophy mice. Shayan and Huang (2020) [40] reported that the therapeutic effect of the MSCs on the de novo myofiber formation requires suitable microenvironmental factors that support the myogenic phenotype. It is therefore urged that before cell delivery the microenvironmental support should be assured in the host considering the disease abnormality [41]. To improve MSCs cell retention and differentiation in the harsh microenvironment, the cells were genetically modified via various methods including viral transduction, gene transfection and pretreatment of modulating factors prior to MSCs implantation [42]. However, a thorough evaluation of the clinical setting is essential as in vitro conditions might not represent the true in vivo milieu. Hence, standards are needed for the clinical use of GM MSCs to ensure the safety, reproducibility and efficiency of the “cell drug” [41].

#### 4.1.3. Amniotic Fluid Stem Cells (AFSCs) 

Considering all the challenges posed by ESCs and adult MSCs, it is worth exploring the potential of fetal-derived stem cells obtained from the human amniotic fluid as an alternative for cell-based therapy. Amniotic fluid stem cells (AFSCs) can be isolated using immunoselection with antibodies specific for c-Kit [43]. AFSCs represent a novel class of pluripotent stem cells with intermediate characteristics between embryonic and adult MSCs stem cells, as they are able to differentiate into lineages representative of all three germ layers but do not form tumors when injected in vivo [30]. Sessarego et al. [44] indicated that no karyotypic abnormalities were observed on the AFSCs tested at different passages despite the extensive proliferation of AFSCs. In contrast, karyotype abnormalities were observed in adult MSCs tested at different passages. Based on the qualities offered by AFSCs such as high proliferative and differentiation potential, low immunogenicity and lack of ethical problems concerning their employment has made the AFSCs a potential novel source for cell-based therapies in VML injury treatment [45]. To date, very limited literature is available on the application of AFSCs for the treatment of muscle injury. A study conducted by Zia et al. [46] showed that AFSCs were able to modulate the expression of specific growth factors involved in muscle regeneration such as transforming growth factors β as displayed by reducing centronucleated fibers and fibrosis. Looking at the potential of AFSCs for VML therapy, few studies on AFSCs biosafety should be carried out mainly on immunologic rejection and tumorigenicity or unexpected differentiation into a non-desired cell type [47]. For instance, Bollini et al. [48] reported that the transplanted human AFSCs spontaneously developed chondro-osteogenic masses in the rat heart. However, they had suggested that the unexpected differentiation could be avoided by induced cardiomyocytes ex-vivo. AFSCs can provide a long-term alternative to restore normal functions of damaged and injured tissues and organs should obstacles such as immunologic rejection and the unexpected differentiation into undesired cell types are successfully overcome. Due to the AFSCs beneficial including pluripotency and the lack of ethical issues associated with human embryonic stem cells research, they should be a promising cell source for regenerative medicine including VML. 

#### 4.1.4. Induced Pluripotent Stem Cells (iPSC)

In 2006, Takahashi [49] had successfully reprogrammed skin fibroblast into a pluripotent stem that mimics the embryonic stem cells. The iPSCs technology is expected to significantly advance research and development in the area of regenerative medicine, pathogenesis and drug screening attributable to iPSCs easy expansion and the ability to maintain their full stem cell potential [50]. Nevertheless, difficulty producing highly purified iPSCs in larger quantities remains the major challenge as cell-based therapies required an ample amount of cells during the treatment [51]. In the case of VML injury, a large number of cells are required as the progress of muscle regeneration is totally depending on the number of cells injected in the area. However, the number of cells population needed to treat VML remains unknown as no data from clinical trials or testing on larger animals available in the literature. Most of the data available were on mice models. Van Der Wal et al. [52] has injected 5 × 10^5^ of iPSCs into mice models with muscle-induced-injury to study the efficacy of iPSCs migration in vivo. They had found that the iPSCs successfully migrated and differentiated into myofiber after intramuscular engraftment in immunodeficient mice in vivo. Moreover, upon optimizing the differentiation process, they have further discovered that the cells expressed fast major histocompatibility complex and skeletal muscle proteins and formed functional sarcomeres (Figure 1) for spontaneous contractions.

### 4.2. Tissue Engineering Scaffolding

Cell therapy’s imminent success could be achieved by employing the right biomaterial cell carrier that can recreate the suitable microenvironment that acts as a scaffold for neo-tissue development and exhibits the activation of the regeneration process [53]. This is particularly true given that each type of stem cells requires its unique encapsulating milieu, with individual material properties and spatially controlled bioactive patterns [54]. To maximize the potential therapeutics utility of cells and growth factors, various studies on scaffolds design and fabrication techniques have been carried out as displayed in Table 1. As discussed in detail above, stem cells hold significant promise to construct implantable functional muscle tissue that could be served as a potential therapeutic agent for treating various skeletal muscle diseases and injuries, such as muscle dystrophies and VML. Among all, bone marrow-derived (MSCs) and iPSCs show encouraging results in treating muscle injury in mice models [55]. However, the ability to control the stem cells differentiation into myofiber with high efficacy and purity remains a formidable challenge. In recent years, the combination of scaffolds and stem cells with suitable biochemical and physiochemical factors has improved stem cells proliferation, while maintaining their plasticity. The functional scaffolds have demonstrated promising results on regulating MSCs differentiation into various mesenchymal phenotypes, such as osteoblast, chondrocytes, myocytes and tendon-ligament fibroblasts without expressing the tumorigenic phenotype of the MSCs [56]. Peng et al. [57] showed that the functional scaffolds could also support the MSCs survival, growth and differentiation even in the absence of stem cell supplementation. To date, considerable effort has been put into the development of functional scaffolds from various fabrication techniques such as solvent casting [58], thermally induced phase separation [59], self-assembly [60], rapid prototyping [61] and electrospinning [62].

## 5. Engineered Natural Polymeric Scaffolds for Muscle Regeneration

A Scaffold serves as a 3D temporary support or matrix that facilitate the cells migration, adhesion, and cells or bioactive molecules transportation that is aimed for tissue repaired or regeneration (ASTM F2150-02, 2002). As an artificial matrix, a scaffold act as a temporary guide or template for cell adhesion, growth and function. The desired engineered scaffolding should offer the following characteristics as summarized by Hoque et al. [72].

Biocompatible
Good mechanical properties to resist cells microenvironment stress
Bioresorbable
Tuneable Degradation
Highly porous to allow cell infiltration and nutrient delivery
Appropriate pore size for cell growth
Conducive surface for cell attachment
Able to functionalize to bioactive signals for favorable cellular interactions.

In this section, recent advances in skeletal muscle tissue scaffolding with the use of electrospun and hydrogels from natural polymers are highlighted.

### 5.1. Electrospun Scaffolds

**Figure 5:**
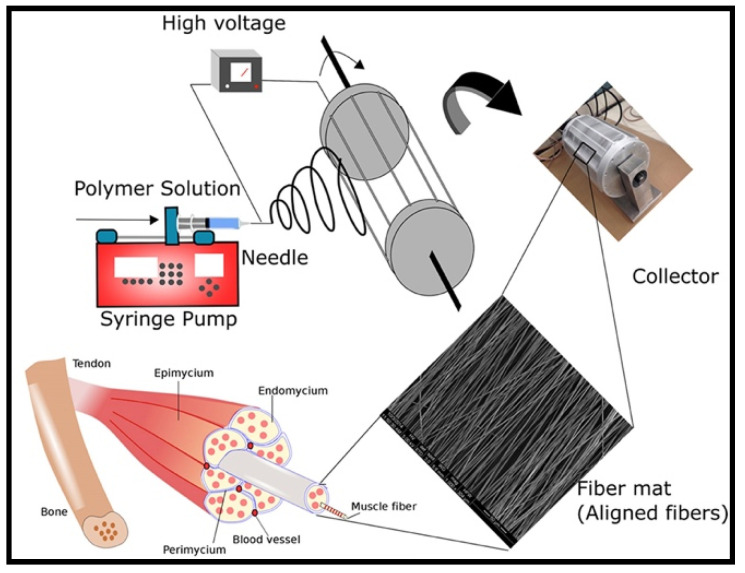
Schematic illustration of electrospun scaffold fabrication for tissue muscle regeneration via electrospinning technique [79]. Copyright © 2020 Frontiers Media SA.

Over the past decade, advances in tissue engineering have sparked interest in biodegradable and biocompatible natural polymers for fabricating tissue scaffolding that mimics the functions of native ECM macromolecules both structure and functions. In this regard, collagen always the best choice as it is a major structural constituent of many tissues such as skin, bone, tendon, ligament and other connective tissues, and accounts for roughly one-third of total body protein [73]. The majority of collagen found in the ECM is in a fibrillar form with long slender filaments around ~67 nm periodicity for structural integrity and strength [74]. More than 27 distinct isotypes of human collagen have been identified [75]. Among the isotypes of collagen, type I and type III are the principal structural components of ECM. Type I collagen is composed of two α1 -chain and one α2-chain. Meanwhile the type III collagen is only composed of three α1 chains that are arranged into a repeating motif that forms a coiled structure [76]. Scientific investigations on collagen have inspired the generation of extracellular matrix analogs for tissue regeneration including VML defects. Collagen types I, II and III have been successfully fabricated into different scaffolds for supporting the growth and function of many cell types [77,78]. To date, electrospinning is the best technique to fabricate a pure collagenous scaffold that mimics the native three-dimensional architecture of the collagen network (Figure 5).

Considerable effort has been made to produce electrospun collagen. Earlier work on collagen electrospinnability was first reported by Matthews et al. [76]. They have successfully spun the collagen type I and III into nanofibers after a series of studies involving the optimization of the working parameters, such as type of solvent, collagen concentration, applied potential and air gap. They also found that structural properties of the electrospun collagen nanofibers vary according to tissue origin and collagen type. For instance, type I collagen from the human placenta exhibits a less uniform matrix of collagen nanofibers compares to calfskin. Matthews et al. [80] have also successfully fabricated collagen type II isolated from chicken sternal cartilage. The in vitro cell culture study demonstrated that the electrospun collagen type II scaffolds promote better cell growth and proliferation compared to type I collagen isolated from human placenta and calfskin. A number of studies on collagen nanofibers efficacy in promoting muscle regeneration were reported. For instance, Joshi et al. [81] reported that the collagenous scaffolds had influenced skeletal muscle differentiation and maturation. This is attributable to their 3D features that mimic the ECM natural polymer with the high surface area available for cell attachment. A systematic study by Witt et al. [82] revealed that incorporating growth factors into the collagen-based scaffolds had enhanced the myogenic differentiation of myoblast.

Another widely used natural polymer is gelatin, a natural biopolymer derived from collagen and has almost identical composition and biological properties as those of collagens [83]. Gelatin is commercially available at a low cost as it can be easily obtained from animal tissues rich in collagen. Due to its natural abundance and inherent biodegradability and biocompatibility in physiological environments, gelatin is widely used in food, cosmetic, pharmaceutical and medical applications [84]. However, the electrospinnability of the gelatin is remained a challenge as gelatin is aggregated in the aqueous environment due to the presence of amino acids with ionizable chains, such as tyrosine, cysteine and arginine [85]. Therefore, highly polar solvents are commonly employed to dissolve the gelatin for better electrospinnability. However, most of the highly polar organic solvents such as hexafluoroisopropanol and trifluoroethanol that are commonly used to fabricate gelatin nanofibrous scaffolds are cytotoxic [86]. Strong hydrogen bonds formed between the gelatin and the organic solvents present a challenge to completely remove the solvents from the scaffolds as the impurity may cause toxic and harmful to the cells. Moreover, the unreacted residuals may also compromise the long-term performance of the scaffolds. Therefore, various acids and their water alcohol mixtures such as formic acid and acetic acid were investigated as a candidate for less cytotoxic solvents for optimum gelatin electrospinnability [62,87].

Gelatin has many distinctive features that confer an advantage in tissue regeneration. However, without proper treatment the gelatin exhibits poor mechanical properties. Post-spinning treatment, such as crosslinking proved to improve water-resistant ability, thermo-mechanical properties and tensile strength. In crosslinking, the aqueous glutaraldehyde reacts to the hydroxyl and sulfide group from amino residues to form strong covalent bonds through intra and intermolecular bonding [88]. Zhang et al. [89] demonstrated that the crosslinking of electrospun gelatin with saturated glutaraldehyde vapor for more than three days had improved the scaffolds tensile strength nearly 10 folds compared to the untreated electrospun. The crosslinked scaffold also exhibited good integrity against the hydrolytic degradation test even after a week of immersion in the PBS. The gelatin scaffold retained 32% of its residual mass after 15 days of incubation [90]. Therefore, crosslinking is an effective technique to improve the mechanical properties of a gelatin-based scaffold [91]. The mechanically robust scaffold is essential in tissue regeneration as it influences cells shape and morphology, protein expression, cell differentiation and tissue organization. Balavigneswaran et al. [92] reported that scaffolds with better stiffness and elasticity similar to the cells microenvironments would enhance cell migration and proliferation. They also found that the gelatin-based scaffold has provided biochemical and physical cues supporting cellular adhesion and cell proliferation.

Besides collagen, fibrin is another fibrillar protein that is naturally involved in wound healing and tissue repair. For years, fibrin has been widely used as hemostatic agents and tissue sealants in various clinical applications including neuro, cardiac and liver surgery. Due to its angiogenic potential, fibrin has been the subject of many studies in the past few years, particularly in volumetric muscle loss injury repair. Recently, Guo et al. [93] have successfully incorporated myoblast into fibrin-based electrospun microfibers via coaxial electrospinning technique. Despite subjected to high voltage during the electrospinning process, the myoblast survived and continued to proliferate. Higher myoblast viability was observed after optimizing the electrospinning parameters. Although higher myoblast density presence within the scaffold, the formation of multinucleated myotubes remained low. Similarly, Gilbert-Hornick et al. [94] reported that poor myogenesis was also observed on the electrospun fibrin microfibers seeded with adipose-derived stem cells (ASCs), a promising cell source with myogenic potential. However, further analysis of the severely injured VML model exhibited that the fibrin fibers loaded with ASCs have integrated well with the native tissue and stimulates cellular and vascular ingrowth. Gilbert-Hornick and friends believed that despite the poor myogenic potential, the fibrin loaded ASCs has successfully promoted moderate muscle reconstruction in severe VML injury.

Cellulose is a renewable polymer available abundantly in plant-based materials. It consists of a linear chain of glucose molecules with a degree polymerization up to ten thousand. The two monomers of anhydroglucose rings joined together by a covalent bond called β 1-4 glucosidic bond to form a linear configuration of the cellulose chain [12]. The chain stabilizes via the interchain hydrogen bonding. The intra and interchain reaction between the adjacent molecules promotes fibrils that are further arranged into larger microfibrils or polysaccharide bundles that are mechanically stable. The outstanding mechanical properties of cellulose has inspired the fabrication of cellulose-based scaffold for muscle regeneration [53]. The electrospun cellulose exhibits high mechanical strength and high elasticity which is desired in accommodating muscle contractions and cell proliferation. The functionalization of electrospun cellulose with vitamin D has further improved, muscle cell migration attributable to the improvement of myotube fusion and differentiation [95]. The vitamin D3 also increased the myotube hypertrophy after 10-days of post damage.

Chitin, poly (*β*-(1-4)-*N*-acetyl-glucosamine) is the principal structural polysaccharide of arthropods (such as crabs and insects) and cell walls of fungi and yeast. Considering the amount of chitin produced annually in the world, it is the most abundant polymer after cellulose [96]. The most important derivative of chitin is chitosan, obtained by partial or fully deacetylation of chitin. The properties of chitin and chitosan such as biocompatibility, biodegradability, antimicrobial activity, non-toxicity and wound-healing property make them suitable candidates for biomedical applications especially in muscle injury scaffolding. Like others, the chitosan’s electrospinnability is not straight forward as its formed colloid in the aqueous medium. At a low pH solution, the chitosan forms dissolution with high viscosity, conductivity and surface tension that lead to bead fibers and poor electrospinnability. Despite all the difficulties, beadles nanofibrous electrospun chitosan has been successfully achieved by blending it with synthetic polymers such as PVA and PEO [97]. However, pure chitosan nanofibers remained a critical research objective as the hydroxyl and amino groups of chitosan involve in the monocyte-based macrophage activation. 

In 2004, Ohkawa et al. [98] for the first time successfully prepared pure chitosan nanofibers with a mean diameter of 330 nm through electrospinning technique. Later, a smaller diameter pure chitosan electrospun with the mean diameter of 70 nm were fabricated by using a 90% acetic acid solution [99]. The functional versatility of the chitosan electrospun fibers, including desirable cell adherence, absorption, oxygen permeability, resorbability and occlusivity has enticed researchers to exploit this material for implants in muscle regeneration. Despite their good qualities, poor mechanical properties always inhibit the application of chitosan in skeletal muscle regeneration as higher modulus and elasticity are required for the muscle cell microenvironments. Since, the muscle cell required high rigidity and elasticity scaffolds, Shim et al. [100] have electrospinned the chitosan onto predefined microfibrous sheets as frameworks. The resulting scaffolds has dual porous architecture containing nanofibrous walls and micro-sized pores formed by a microfibrous sheet. The nano/microfibrous 3D matrix offered an excellent stiffness, thus provided a great microenvironment for muscular cell cultivation and proliferation. Recently, adipose-derived stem cells seeded on the chitosan-based scaffolds have demonstrated muscle regeneration and served as a bridge to connect to the injured muscle [101]. The adipose-derived stem cells are believed to be highly involved in promoting the muscle’s regeneration, which includes differentiation, migration, and paracrine effects. In other studies, Ronchi et al. [102] found that the chitosan based scaffolds have promoted both muscle and nerve regenerations. They found that higher expression of Neuregulin 1 by the skeletal muscle tissue seeded on the chitosan-based scaffolds has triggered the stimulation of Schwann cell survival and promoting axon growth of the injured nerve.

Similarly, hyaluronic acid has also been reported to enhance repair in damaged nerve and skeletal muscle. The hyaluronic acid promotes the high release of myogenic regulatory factors that facilitate the functional repair of mammalian skeletal muscle in vivo. However, their potential in nanofiber electrospun has not been fully exploited. This is because hyaluronic acid shows poor processability during electrospinning due to polyelectrolyte nature that caused high solution viscosity and surface tension at very low polymer concentrations. To reduce the polyelectrolyte effect, Li et al. [103] have electrospun hyaluronic acid in DMF and water with an elevated environmental temperature (40 ± 3 °C). An ethanol bath was accommodated to prevent fusion of electrospun nanofibers on the collector, since hyaluronic acid exhibited strong water retention ability. A highly basic solvent system was investigated for the successful electrospinning of hyaluronic acid by incorporating sodium hydroxide (NaOH) with DMF solvents [104]. However, a highly basic solvent system may detrimental to electrospun hyaluronic acid mechanical properties. Brenner et al. came up with a less basic solvent system combining aqueous ammonium hydroxide (NH_4_OH) and DMF. This system reached a pH of 11, compared to 13 of the NaOH/DMF system. The average nanofiber diameter of 39 ± 12 nm was obtained when 1.5% hyaluronic acid dissolved in 2:1 (*v*/*v*) NH_4_OH: DMF solutions. Besides basic solvents, an alternative acidic solvent system could also generate electrospun hyaluronic acid nanofibers. A system consists of formic acid, water and DMF was studied [105]. As suggested by the authors, the addition of formic acid could disrupt the rigid alpha-helix of hyaluronic acid by reconstruction hydrogen bonds to hyaluronic acid molecules. A modified electric field with the aid of a customized collector was also achieved. A mountain-folded aluminium foil was used to achieve a distorted electric field. The charge would accumulate on the crests of the collector and close to zero on the concaves. The as-spun nanofiber exhibited a diameter range between 30 to 50 nm, however, the nanofiber morphology was not uniform.

### 5.2. Hydrogels

Over the past decades, hydrogels have revolutionized the biomedical field and have been employed in a wide variety of applications including in muscle regeneration especially after volumetric muscle loss. The hydrogel is a 3D network polymer that is made of crosslinked natural or synthetic materials that can swell yet maintained its mechanical integrity. The ability to swell under biological conditions and good mechanical properties from the crosslinking makes them an ideal candidate in tissue engineering and drug delivery. Hitherto, two techniques are commonly applied to muscle regeneration using hydrogels including injectable hydrogels and pre-formed hydrogels-based scaffolds. The latter technique is widely used to guide in vitro muscle tissue formation or to orchestrate in situ skeletal muscle regeneration [106]. Naturally derived hydrogels remain the best option as the hydrogel provides cell adhesion and exhibits strong cells interaction. In contrast, the synthetic and hybrid hydrogels are always associated with non-specific interaction with the cells and correlate to proteolytic degradation on the hydrogels.

Recently, increasing efforts have been made to design and fabricate natural polymeric based hydrogels due to their similar structure to components in the body. Moreover, they elicit a limited inflammatory response and exhibit adequate biocompatibility for tissue regeneration [107]. Among the natural polymeric materials studied, it was found that fibrin demonstrated greater potential to support activation, proliferation and differentiation of primary murine satellite cells as fibrin appears to mimic the satellite cell niche. To further increase myoblast proliferation and pro-regenerative growth factor secretion, Marcinczyk et al. [108] have enriched the fibrin hydrogels with laminin. The in vitro myoblast seeded on the fibrin-based hydrogels enriched with laminin exhibited improvement in cells elongation, alignment and myokine secretion after 24 h of post-stimulation. To improve myoblast survival and differentiation within the scaffolds, Matthias et al. [109] explored the potential to employ directly in situ defect casting using fibrin hydrogels seeded with myoblast. The method has allowed significant muscle mass restoration and fibrosis reduction with the active contribution of transplanted cells in the muscular and vascular regeneration. Scaffolds with good mechanical properties are highly desired as muscular-skeletal muscle tissue requires strong and elastic scaffolds to resist the mechanical forces exerted by the cells. Wu et al. [110] has designed protein-based hydrogels from short protein building blocks with predictable mechanical properties. The predictable scaffolds mechanical properties would be useful for specific tissue engineering application that requires certain mechanical features. In the study, they also found that long and flexible proteins are unpredictable due to the entanglement of different protein chains and random physical crosslinking that would change the macroscopic mechanical properties. Moreover, non-specific intermolecular interaction is another challenge in fabricating hydrogels with predictable mechanical properties. In most cases, the non-specific interaction can lead to mesoscopic clusters of protein chains that eventually lead to protein unfolding and aggregation, making the prediction difficult.

**Figure 6:**
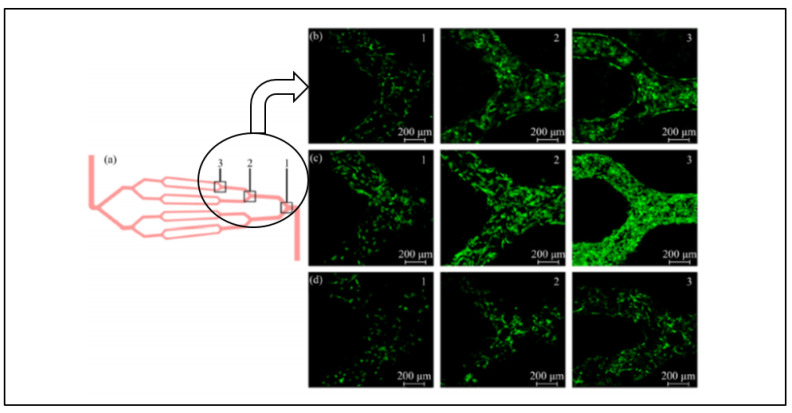
Culture of the cells in a vessel-like network (**a**). Model predefined microchannels, (**b**–**d**) phase images of cell morphology inside three-level channels after cultivation for four days, seven days and 14 days [113]. Reprinted by permission of springer Nature. Copyright © 2016 by Springer Nature.

Other than fibrin, gelatin is one of the most attractive protein-based candidates for consideration as biomaterial scaffolds in muscle tissue regeneration. Recently, Gattazzo et al. [111] has successfully fabricated gelatin-based hydrogels crosslinked with genipin to improve the mechanical properties so it could mimic skeletal muscle mechanical properties. It was reported that the scaffolds stiffness exhibits highly potent regulator for cells proliferation and differentiation into myotubes, an essential precursor for skeletal muscle regeneration. They had also observed high presence of inflammatory cells including macrophages at the animal models. The high presence of macrophages at the gelatin hydrogel implants triggers the inflammatory cascade activation and eventually stimulates the satellite cells proliferation and differentiation for myofibers formation. To further influence the seeded cell behavior, Tijore et al. [112] has proposed 3D bioprinting gelatin hydrogels. The bioprinted gelatin hydrogel has established cell-cell contacts all across the scaffolds. Direct interaction between cell is critical to the development and function of multicellular organisms [15]. The most recent development in hydrogel scaffolds was the fabrication of multi-level vessel-like networks in the enzymatic gelatin hydrogels. The branched and multi-level microchannel network developed by Dong et al. [113] simulates biomimetic microenvironments of the cellular ECM that effectively and efficiently promote tissue regeneration. The network construct exhibits great promise in developing ECM and organ assisting devices in muscle regeneration as the nutrients and oxygens can be easily supplied to the cells via microfluidic vascular. The ability to encapsulate the oxygen diffusion via the vessel-like channel would certainly be desirable for promoting and supporting myoblast proliferation in volumetric muscle loss injuries. Figure 6 depicts the cells seeded on the vessel like a network.

The exploration of self-healing hydrogel has gained immense attention especially by the exploitation of dynamic covalent bonds present in polysaccharides, such as imine bonds that can be prepared between amino and aldehyde groups under physiological conditions [114]. Chitosan is highly anticipated as an excellent candidate for self-healing hydrogels due to the presence of glucosamine and N-acetyl glucosamine on its backbone [115]. Nevertheless, high quality self-healing chitosan-based hydrogel could only be attained with the presence of proper crosslinking agents. Jing et al. [116] have exploited dopamine for the formation of covalent bonds and hydrogen bonds with chitosan to provide physically and chemically crosslinked networks. Moreover, the addition of dopamine also enhanced adhesive properties. They also found that the skeletal muscle cells exhibited a double proliferation rate after seeding on the chitosan-based hydrogel compared to the normal petri dish. One of the major problems in self-healing hydrogels is their poor mechanical properties such as low strength and toughness. Many efforts have been made to improve the mechanical properties including the addition of nanomaterials [117]. In their study, the authors developed self-healing hydrogels based on polysaccharide biopolymers doped with graphene to improve the crosslinking efficacy. The addition of graphene has induced the formation of a strong and dynamic hydrogen bonding between the agar. Furthermore, the homogenous dispersion of the graphene within the matrix further favoured the interfacial interaction. Stronger self-healing hydrogel has been developed by Wang et al. [118] from chitosan with the presence of ferric cation sprouting metal-coordination and chain entanglement. The scaffolds can resist high fracture stress of 1.4 MPa as well as elongation at break of about 700% after healing. The impressive properties of the hydrogel make it a better candidate for providing temporary support for skeletal muscle cells.

### 5.3. Acellular Scaffolds

Biological scaffolds derived from de-cellularized tissues and organs are progressively applied in regenerative medicine and tissue engineering [119]. The technology uses a thermo-responsive substrate to generate artificial conduits containing sheets of cells embedded in a native ECM [120]. The biological conduits offered a non-immunogenic allogenic scaffold containing guidance cues present in the tissue regeneration. This method has successfully produced cell sheet technology for cells deposition from various cells isolated from cornea [121], areolar [122], lung [123] and heart [124]. Acellular tissues, such as pig urinary bladder ECM, have been clinically used to treat VML conditions [125]. The results of the 13-patients cohort study show that an acellular biological scaffolds approach has facilitated constructive and functional tissue remodelling on the VML patients. The use of ECM scaffolds appears to mediate myogenic progenitor cells migration, improved innervation and functional skeletal muscle formation. Recently, attempts have been made to use acellular muscle to promote functional muscle regeneration without the implementation of donor cells [126]. They have found that all the acellular scaffolds implanted on the resected mice’s muscles were able to generate functional artificial muscles by promoting host myogenic cell migration and differentiation, as well as nervous fibres, vascular networks and satellite cells homing. As compared to the previous study, no donor cell implementation was needed. Therefore, less time is required in the therapeutics as no requirement for cultivating donor cells prior to the implantation.

In another work, Urciuolo et al. [127] have highlighted that the large volume of tissue that needs to be regenerated in patients affected by VML can be a major limiting step for clinical application of acellular tissue. The first in vivo studies on larger animals as a model for VML injury was conducted by Turnet et al. [128]. They have used a dissected canine VML model and the injury was implanted with acellular scaffolds from porcine small intestinal submucosa. The initial remodeling process exhibited a similar pattern reported in previous studies that ECM-mediated muscle repair with rapid vascularization and migration of myoblast into the defect site. After long-term implantation, the scaffold promotes the formation of dense collagenous tissue with island of muscle within the segments of the scaffold. Despite the formation of the collagenous tissue, no successful restoration of muscle functionality was observed.

A study by Svystonyuk et al. [129] revealed that the amount of essential growth factors retain in the biological scaffolds may not be sufficient to enable the production of functional human muscle tissue construct. The aggressive decellularization process including exposure to non-physiologic chemical and biological agent such as detergents and enzymes may attribute to degradation of essential growth factors and structural proteins [130]. Chen et al. [131] has immobilized the growth factor on the acellular scaffold to maintain local therapeutics dosage at the targeted site. The presence of high primary amines and carboxyl groups on the acellular scaffolds makes the conjugation of growth factors easier with the presence of crosslinkers, such as carbodiimide and succinate ester. The in vivo studies by Chen and colleagues revealed that the growth factor targeted delivery system shows promising results on animal models with induced tissue injury. Nakayama et al. [132] reported that growth factors such as vascular endothelial growth factors (VEGF), nerve growth factor (NGF) and glial-derived neurotrophic factor (GNDF), which are essential in functional muscle development have been successfully immobilized and covalently conjugated onto biological scaffolds. However, rapid degradation and loss of bioactivity are among the major obstacles and limitations of the approach [132]. Recently, different techniques have been developed including physical coating [133] and core-shell electrospun [134] to improve the growth factors bioactivity.

### 5.4. D Bioprinting

In recent years, researchers have focused on mimicking the ultrastructure of native muscle tissue composed of highly oriented myofibers [135]. This is because it has been proven that functional muscle construct could be attained by controlling spatial organization of multiple myofibers bundles [136]. In the previous section, successful attempts on controlling muscle cells orientation and alignment in a uniform direction have been highlighted. However, the strategies were only allowed micro-scale tissue or single-layered muscle bundle construction which successfully improve muscle cells functionality in vitro but maybe not suitable for treating extensive muscle defects. The advances in additive manufacturing especially in 3D bioprinting has attracted attention especially in the fabrication of complex scaffolds geometry to bioengineer functional muscle construct for treating volumetric muscle loss (VML). Controlling organization of bioengineered muscle tissue in vitro should be essential for functional tissue restoration after implantation in vivo. Therefore, the ability to recapitulate the organization and function of the native skeletal muscle remains the most important element in bioengineered skeletal muscle.

**Figure 7:**
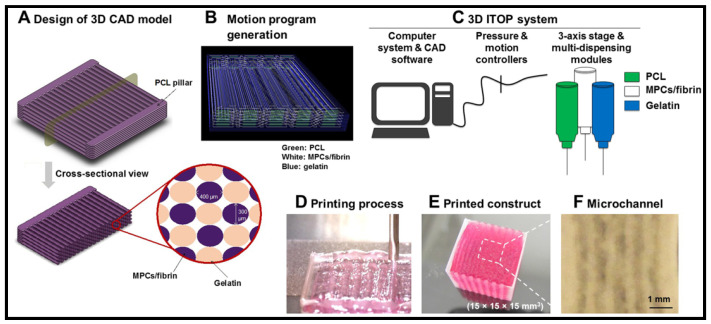
3D bioprinted human skeletal muscle construct for muscle function restoration [136]. Reprinted by permission of springer Nature. Copyright © 2018 by Springer Nature.

Kim et al. [136] present a novel bio-printing technique, using integrated tissue organ printing and natural bio-ink (fibrinogen/gelatin/hyaluronic acid), in order to generate a 3D freeform shape architecture that can construct organized muscle structure similar to a native muscle (Figure 7). They reported that the bioprinted construct has successfully accelerated human muscle progenitor cell (hMPC) maturation and differentiation into highly viable, densely packed and spatially aligned myofibers, in vitro. The animal test evaluation demonstrates that the implant has restored muscle functions. Highly organized vascularize muscle tissue and nerve integrity were observed in the rat model with severe VML injury. Kim and colleagues believed that full restoration of muscle functions following VML injury could be achieved by improving vascular network presence on the muscle construct. In different attempts, Bou et al. [137] reported that network-like structures could be attained by printing endothelial cells (EC) and myoblast on the acellular bladder matrix. The co-culture bioprinting technique has enhanced the formation of pre-vascularized microvascular networks on the scaffold. Kuss et al. reported that a high presence of angiogenic factors, such as vascular endothelial growth factors and basic fibroblast growth factors were also observed on the co-culture construct [138,139]. The angiogenic factors are important in maintaining microvascular integrity and stability, which eventually promotes muscle construct maturation [122]. As development progresses, clinically useful muscle construct with adequate force to restore muscle function can be accomplished by this approach.

## 6. Challenges and Future Perspectives

The field of volumetric muscle loss injury and repair is an exciting one. Many research groups are attempting to design new and successful strategies to repair and reconstruct the artificial muscle. The natural based scaffold has proven to be an extremely useful approach to regenerate muscle loss by promoting myoblast migration [140]. Despite the excellent performance of natural polymer-based scaffolds during the in vitro analysis, the in vivo approach for VML remain a major challenge especially on the larger animals and human patients. Thus far, the results on larger animals show poor restoration of muscle functionality. Missing or insufficient amount of paracrine hormones in situ could be the reason for poor myoblast migration [141]. Therefore, the incorporation of the paracrine hormones could be the best option to cater the problem. Other concerns on employing the natural polymeric scaffolds are the poor mechanical strength and batch variation [142]. In this review, we have illustrated various techniques by other researchers on improving the mechanical properties of natural based scaffolds. As depicted in the previous section, materials with excellent mechanical properties are highly desired to cultivate the skeletal muscle cells which exhibited higher mechanical microenvironments [143]. The exploration of safe and more effective crosslinkers is highly desirable to improve the mechanical properties of the scaffolds. Currently used crosslinkers such as genipin, glutaraldehyde and etc. are very effective in improving the mechanical properties of the scaffolds [144]. However, concern about toxicity always hinders their application in clinical application. Physical crosslinking is worth exploring. Nevertheless, extensive and thorough studies are needed to optimize the efficacy of the scaffolds for both in vitro and in vivo application. Batch to batch variation is another major drawback of employing natural polymers. Variability from batch to batch of the scaffolds, fabricated from natural polymers, is inevitable. Moreover, batch to batch variation may also provoke an immune response when implanted in vivo due to the xenogenic polymer source [145]. The synthesis of directed short chain polysaccharides and polypeptide via polycondensation or classical solution phase peptide synthesis could be the solution to control the batch variation in natural based scaffolds. Moreover, mutagenesis could also be the solution for batch variation. The directed synthesis of the natural polymer would also eliminate the concern of complication from zoonotic pathogen infection.

In vivo energy generation and internal charging by the natural body activity and the physiological environment has played a vital role in cell division, intracellular communication, neural activities, epithelial healing and mechanotransduction [146]. Electrotherapy that manipulates exogenous electric current has shown to be a promising treatment option for accelerated wound healing, improved skeletal muscle repair and tissue regeneration. Various external devices such as conductive coupled monopolar and coupled bipolar made of polymeric and carbon base materials have been developed to supply low level exogenous electric currents at the site injury [147]. Nevertheless, the complexity and inconvenience to patients associated with electrotherapy have triggered the development of scaffolds that emulate biological electricity for tissue regeneration without an external source of electrical stimulation or electrodes’ implantation [148]. Scaffolds made of piezoelectric biomaterials are an attractive choice as they exhibit electromechanical behavior that transforms mechanical energy into electric polarization without external voltage. Such piezoelectric biomaterials offer numerous advantages over conventional biomaterials as they can easily transduce electricity to living systems in response to processes such as body movements and cell migration. Pizopolymers and piezoceramics have been widely used for different tissue repair applications, particularly in bone repair, where mechanical stress charges have enhanced bone formation [149]. Despite the demonstrated potential in tissue regeneration, there are just a few conclusive works addressing the effect of electrical stimulus promoted by piezoelectric on muscle regeneration. It is speculated that the electrical and mechanical stimuli transduced by piezoelectric biomaterials may induce myogenic differentiation and functional maturation of muscle like tissue [150].

## Figures and Tables

**Table 1:** Summary of common techniques to fabricate nanofibrous scaffolds for tissue engineering applications.

Technique	Advantages	Disadvantages	Example	Ref.
Solvent casting/Particulate Leaching techniques	Control over porosity, pore size and crystallinity	Use of highly toxic solvents Labor intensive fabrication process Residual particles in the polymer matrix Irregular shaped pores Insufficient interconnectivity	PLGA *^a^/*Gelatin as scaffolds for cell-based artificial organs. **Findings:** Enhanced cells adhesion and proliferation of chondrocytes and smooth muscles.	[58]
Gas foaming	Free of harsh organic solvent Control over porosity, pore size and fiber diameter	Formation of a non-porous matrix resulted from rapid diffusion of gas away from the surface Lack of interconnectivity between pores.	PCL *^a^*/Gelatin as scaffolds for new tissue regeneration **Findings:** The human mesenchymal stem cells (hMSCs) were able to colonize the outer and inner regions of the scaffolds.	[63]
Thermally-induced Phase separation/Porogen leaching	Versatile Control over pore size when combined with other techniques Great control over the 3D shape	Little control over fiber diameter and orientation Time-consuming	Pure gelatin based scaffold for Tissue engineering applications **Findings:** The 3D shape with porous and nanofibrous scaffolds has induced a higher level of osteocalcin and bone sialoprotein expression (bone markers).	[59,64]
Wet spinning	Large surface area for cell attachment and rapid diffusion of the nutrients in favor of cell survival and growth	Poor mechanical properties.	Chitosan based scaffolds for bone tissue engineering **Finding:** The scaffolds allowed significant cell proliferation of osteoblast and exhibited good attachment and developed bridging between cells via filopodia structures.	[65,66]
Fiber bonding	Produce highly porous scaffolds with interconnected pores	The solvent used could be toxic to the cells if not completely remove	PGA *^a^*/PLLA *^a^* as polymeric scaffolds for Cell-based artificial liver **Findings:** A higher degree of interaction between hepatocytes and porous scaffolds after 18 hours of cultivation. Major interaction between cell-cell rather than cell-polymer was observed after 1week of cultivation.	[67,68]
Self-assembly	The scaffolds can be modified. Do not produce synthetic degradation by-products The scaffolds provide the opportunity to incorporate modified variants containing quite large bioactive motifs or domain	Expensive material and complex design parameters.	Peptide as natural based scaffolds for promising scaffolds for the study of cell signal pathway **Findings:** The functionalized peptides that underwent self-assembly into nanofiber structures have significantly enhanced the neural cell survival without additional extra growth factors.	[60]
Rapid prototyping	Produce scaffolds with a fully interconnected pore structure. Full control over porosity, pore size, pore shape and permeability.	Highly expensive equipment	HA *^a^*/PCL *^a^* as scaffolds for bone tissue engineering **Findings:** The high surface area of the scaffolds favors the adhesion and growth of the osteoblast.	[69]
Electrospinning	Inexpensive. Simple set-up. High surface area to volume ratio. Ease of fiber functionalization.Ease of material hybridization. Possibility of scaling–up the process for mass production.	The solvents used could be toxic to the cells if not completely removed. The process depends on many variables.	PCL *^a^*/Gelatin hybrid scaffolds for peripheral nerve regeneration **Findings:** The scaffolds offered a more mimicking micro and macro environment for peripheral nerve regeneration by providing excellent substrate delivery to guide axons regeneration.	[70,71]

*^a^* Abbreviations: PLGA, Poly(lactic-*co*-glycolic acid); PCL, Polycaprolactone; PGA, Poly(glycolic acid); PLLA, Poly(L-lactic acid); HA, Hydroxyapatite.

## Data Availability

Not applicable.
